# Determination of *EGFR* and *KRAS* mutational status in Greek non-small-cell lung cancer patients

**DOI:** 10.3892/ol.2015.3600

**Published:** 2015-08-12

**Authors:** EIRINI PAPADOPOULOU, NIKOLAOS TSOULOS, ANGELIKI TSIRIGOTI, ANGELA APESSOS, KONSTANTINOS AGIANNITOPOULOS, VASILIKI METAXA-MARIATOU, KONSTANTINOS ZAROGOULIDIS, PAVLOS ZAROGOULIDIS, DIMITRIOS KASARAKIS, STYLIANOS KAKOLYRIS, JUBRAIL DAHABREH, FOTIS VLASTOS, CHARALAMPOS ZOUBLIOS, AGGELIKI RAPTI, NIKI GEORGATOU PAPAGEORGIOU, DIMITRIOS VELDEKIS, MINA GAGA, GERASIMOS ARAVANTINOS, VASILEIOS KARAVASILIS, NAPOLEON KARAGIANNIDIS, GEORGE NASIOULAS

**Affiliations:** 1 GeneKor, Athens 15344, Greece; 2 Pulmonary Department, Oncology Unit, ‘G. Papanikolaou’ General Hospital, Aristotle University of Thessaloniki, Thessaloniki 54124, Greece; 3 Oncology Unit, General Hospital of Kavala, Kavala 65500, Greece; 4 Department of Medical Oncology, University General Hospital of Alexandroupolis, Evros 68100, Greece; 5 Department of Thoracic Surgery, Athens Medical Center, Athens 30606, Greece; 6 University Clinic of Pulmonology, ‘Sotiria’ Chest Diseases Hospital, Athens 11527, Greece; 7 Department of Oncology, Evaggelismos Hospital, Athens 10676, Greece; 8 Second Pulmonary Clinic, ‘Sotiria’ Chest Diseases Hospital, Athens 11527, Greece; 9 Fifth Pulmonary Clinic, ‘Sotiria’ Chest Diseases Hospital, Athens 11527, Greece; 10 Department of Respiratory and Critical Care Medicine (KAA), ‘Sotiria’ Chest Diseases Hospital, Athens 11527, Greece; 11 Seventh Pulmonary Clinic, ‘Sotiria’ Chest Diseases Hospital, Athens 11527, Greece; 12 Second Department of Medical Oncology, ‘Agii Anargiri’ Cancer Hospital, Athens 11524, Greece; 13 Department of Medical Oncology, Medical School, Aristotle University, Papageorgiou Hospital, Thessaloniki 54124, Greece; 14 Second Department of Respiratory Medicine, Sismanoglio-A. Fleming General Hospital of Attiki, Athens 15126, Greece

**Keywords:** non-small-cell lung cancer, *epidermal growth factor receptor*, *Kirsten-rat sarcoma oncogene homolog*, high-resolution melting curve analysis, Sanger sequencing, next generation sequencing

## Abstract

It has been reported that certain patients with non-small-cell lung cancer (NSCLC) that harbor activating somatic mutations within the tyrosine kinase domain of the *epidermal growth factor receptor* (*EGFR*) gene may be effectively treated using targeted therapy. The use of EGFR inhibitors in patient therapy has been demonstrated to improve response and survival rates; therefore, it was suggested that clinical screening for EGFR mutations should be performed for all patients. Numerous clinicopathological factors have been associated with EGFR and *Kirsten-rat sarcoma oncogene homolog* (KRAS) mutational status including gender, smoking history and histology. In addition, it was reported that EGFR mutation frequency in NSCLC patients was ethnicity-dependent, with an incidence rate of ~30% in Asian populations and ~15% in Caucasian populations. However, limited data has been reported on intra-ethnic differences throughout Europe. The present study aimed to investigate the frequency and spectrum of EGFR mutations in 1,472 Greek NSCLC patients. In addition, KRAS mutation analysis was performed in patients with known smoking history in order to determine the correlation of type and mutation frequency with smoking. High-resolution melting curve (HRM) analysis followed by Sanger sequencing was used to identify mutations in exons 18–21 of the EGFR gene and in exon 2 of the KRAS gene. A sensitive next-generation sequencing (NGS) technology was also employed to classify samples with equivocal results. The use of sensitive mutation detection techniques in a large study population of Greek NSCLC patients in routine diagnostic practice revealed an overall EGFR mutation frequency of 15.83%. This mutation frequency was comparable to that previously reported in other European populations. Of note, there was a 99.8% concordance between the HRM method and Sanger sequencing. NGS was found to be the most sensitive method. In addition, female non-smokers demonstrated a high prevalence of EGFR mutations. Furthermore, KRAS mutation analysis in patients with a known smoking history revealed no difference in mutation frequency according to smoking status; however, a different mutation spectrum was observed.

## Introduction

Lung cancer is the primary cause of cancer-associated mortality worldwide (1,2). The most prominent etiology of lung cancer is smoking, which is responsible for 80% of cases (3). In addition, non-small cell lung cancer (NSCLC) accounts for ~85% of lung cancer cases (2). The disease is usually diagnosed at advanced stage, resulting in poor overall survival rates. Treatment options for lung cancer include surgery, radiation therapy and chemotherapy (4). Although chemotherapy remains an important form of treatment, novel drug development has focused on molecular targeted therapies, which may enable the use of specific treatments based on a tumor's genetic alterations. The most common somatic mutations in NSCLC are located in the *epidermal growth factor receptor* (*EGFR)* and *Kirsten-rat sarcoma oncogene homolog* (*KRAS)* genes (5,6).

One of the first molecules successfully used as a target for molecular therapies was *EGFR*. The application of ﬁrst-generation tyrosine kinase (TK) inhibitors (TKI) Geﬁtinib and Erlotinib was demonstrated to confer improved response and survival outcomes in patients with mutations in the TK domain of the *EGFR* gene (exons 18–21) (7–10).

Numerous clinicopathological factors have been associated with *EGFR* and *KRAS* mutations, including gender, smoking history and histology (11,12). In addition, it was reported that *EGFR* mutation frequency in NSCLC patients was ethnicity-dependent, with an incidence rate of ~30% in Asian populations and ~15% in Caucasian populations. However, limited data has been reported on intra-ethnic differences throughout Europe.

*KRAS* mutations are also present in a high percentage of NSCLC patients and are associated with poorer prognosis and resistance to EGFR-TKIs. However, the extent to which this may influence treatment selection remains to be elucidated (13–15). In addition, *KRAS* mutation frequency and mutation spectrum have been suggested to be influenced by smoking habits (16).

Current guidelines recommend testing all patients with metastatic NSCLC adenocarcinomas for the presence of activating *EGFR* mutations; in addition, these guidelines suggest the use of EGFR-TKIs as first-line therapy in patients with adenocarcinoma and a known *EGFR* mutation (17). Thus, accurate mutation detection is crucial for appropriate treatment selection. The most commonly used method for *EGFR* mutation testing was considered to be Sanger sequencing (18,19). However, this method has various disadvantages, since it is considered a laborious technique with limited sensitivity. Thus, this method may lead to false negative results when the mutation percentage or the tumor cell content in the material used is low.

In order to resolve these issues, a variety of methods are currently available for *EGFR* mutational testing. These methods include quantitative polymerase chain reaction (PCR)-based assays, pyrosequencing, high-resolution melting curve (HRM) analysis and peptide nucleic acid-PCR clamp, denaturing high-performance liquid chromatography and next-generation sequencing (NGS) assays (18). These methods all have different advantages and disadvantages; therefore, the use of multiple techniques for *EGFR* mutation testing may increase *EGFR* testing accuracy. In addition, when biased results are obtained from one method, the use of an alternative method may be useful in order to confirm the presence of a mutation. The aim of this study was to determine the frequency and spectrum of *EGFR* mutations in a group of Greek NSCLC patients. Additionally, *KRAS* mutation analysis was performed in patients with known smoking history to determine the correlation of type and mutation frequency with smoking.

## Materials and methods

### 

#### Patients

A total of 1,472 tumors from Greek patients with newly diagnosed NSCLC were analyzed for mutations in EGFR exons 18, 19, 20 and 21. All available clinical factors, including age, gender, histology and smoking history, were evaluated. The age of diagnosis was known for 1,046 patients, pathological reports were available for 497 patients and smoking history was available for 561 patients. Based on their smoking status, patients were categorized as non-smokers (<100 cigarettes in their lifetime), ex-smokers (quit ≥5 year ago) or smokers (quit <1 year ago). For the 561 with known smoking history, KRAS exon 2 analysis was also performed. Informed consent was obtained from all patients prior to testing. This study was approved by the ethics committee of ‘Agii Anargiri’ Cancer Hospital (Athens, Greece).

#### DNA extraction and mutation analysis

DNA extraction was performed using 10-µm-thick sections of formalin-fixed and paraffin-embedded (FFPE) tissue samples. For all samples, pathological review and macro-dissection were performed in order to confirm a tumor cell content of >75%. The tumor area was determined through comparison with the corresponding hematoxylin and eosin stained slide. A NucleoSpin Tissue kit (Macherey-Nagel, Düren, Germany) was used for DNA extraction according to the manufacturer's instructions.

*EGFR* exons 18, 19, 20 and 21, as well as *KRAS* exon 2 mutation analysis were performed using HRM analysis. HRM is a sensitive scanning method used for rapid and reliable mutation screening in human cancers. PCR cycling and HRM analysis were performed on the Rotor-Gene 6000™ (Corbett Research, Mortlake, Australia). The intercalating dye used was SYTO 9 (Invitrogen Life Technologies, Carlsbad, CA, USA). In brief, PCR assays were performed in a 25-µl reaction volume containing 100 ng genomic DNA, 1X PCR buffer (Qiagen Inc., Valencia, CA, USA), 2.5 mmol/l MgCl_2_ (Qiagen Inc.), 200 nmol/l each primer (Invitrogen Life Technologies), 200 µmol/l each deoxynucleotide (New England Biolabs, Inc., Ipswich, MA, USA), 5 µmol/l SYTO 9 (Invitrogen Life Technologies), 1.25 Units HotStarTaq (5 U/µl; Qiagen Inc.) and PCR grade water (Invitrogen Life Technologies).

Primers for all exons were previously described (19,20) except for the reverse primer of the *EGFR* exon 20, which was designed using the primer-BLAST software (http:www.ncbi.nlm.nih.gov/tools/primer-blast). The PCR conditions were as follows: Initial denaturation at 95°C for 15 min, followed by 40 cycles of 15 sec at 95°C, 10 sec at 68–58°C (decrease of 1°C/cycle for the first 10 cycles) and 30 sec at 72°C. For the HRM melting profile, samples were denatured with an initial hold at 95°C for 1 sec and a melting profile from 72–95°C rising by 0.2°C every 1 sec. All HRM reactions were performed in triplicate.

#### Sequencing analysis

In order to perform the Sanger sequencing reaction, a NucleoFast® 96 PCR Clean-up kit (Macherey-Nagel GmbH and Co., Düren, Germany) was used, according to the manufacturer's instructions, to purify the PCR amplification products. Subsequently, 7 µl purified product was used for each sequencing reaction, which was performed using the BigDye® Terminator v1.1 Cycle Sequencing kit (Applied Biosystems, Foster City, CA, USA). Sequencing reaction products were purified prior to electrophoresis using the Montage™ SEQ_96_ Sequencing Reaction kit (EMD Millipore Corp., Billerica, MA, USA). Sequencing analysis was performed on an Applied Biosystems 3130 Genetic Analyzer (Applied Biosystems).

#### Targeted NGS assay

TruSeq Custom Amplicon Library Preparation (Illumina, Inc., San Diego, CA, USA) allows targeted sequencing of the genomic regions spanning upwards of 600 kb with up to 1,536 amplicons in a single multiplex reaction.

A pool of custom upstream and downstream primers were designed using the web-based sequencing assay design tool Design Studio (Illumina, Inc.) (http:designstudio.illumina.com/). The oligos were specific for amplification of specific regions involved in somatic mutations in different types of cancer (Table I). In total, 17 targets were amplified, using 42 amplicons, according to the manufacturer's protocol.

In brief, the custom oligos pool was hybridized to genomic DNA samples. The excess of unbound oligos was removed from genomic DNA using a filter suitable for size selection (Illumina, Inc.). Three additional wash steps ensured complete removal of unbound oligos and prepared samples for the extension-ligation step. During the extension-ligation step the hybridized upstream and downstream oligos were connected. This was achieved using a DNA polymerase (Illumina, Inc.) that extended from the upstream oligo through the targeted region, followed by ligation to the 5′ end of the downstream oligo using a DNA ligase (Illumina, Inc.). The extension-ligation resulted in the formation of products containing the targeted regions of interest flanked by sequences required for amplification. The extension-ligation products were amplified using primers that add sample multiplexing index sequences (i5 and i7) as well as common adapters required for cluster generation (P5 and P7) (Illumina, Inc.).

Subsequently, the PCR products were purified from the other reaction components using Agencourt AMPure XP PCR purification system (Beckman Coulter, Inc., Brea, CA, USA) and the quantity of each library was normalized using normalization additives, beads and wash solution (Illumina, Inc.) to ensure more equal library representation in the pooled sample. Finally, equal volumes of normalized library were combined, diluted in hybridization buffer (Illumina, Inc.) and heat denatured at 96°C for 2 min prior to sequencing on the MiSeq sequencer (Illumina, Inc.). NGS data analysis was performed using the genomics computing environment VariantStudio version 2.2 (Illumina, Inc.).

#### Sensitivity

The sensitivity test was performed using genomic DNA reference standards with defined allelic frequencies (Horizon Diagnostics, Cambridge, UK).

DNAs heterozygous (allele frequency, 50%) for *EGFR* mutations p.L858R (exon 21) and A746-E750del (exon 19) were diluted with wild-type DNA in order to obtain a mutant to wild-type allelic ratio of 50, 12.5, 7.5 and 5%. These samples were used to determine the sensitivity of the HRM and sequencing methods. Calculation of NGS sensitivity, was performed using two *EGFR* Multiplex Reference Standards (Horizon Diagnostics) that cover mutations at codons 719 (p.G719S), 746–750 (A746-E750del), 790 (p.T790M), 858 (p.L858R) and 861 (p.L861Q) spanning exons 19, 20 and 21. These standards were manufactured using five engineered *EGFR* mutant cell lines (Horizon Diagnostics) and mixed to generate 5 and 1% mutant *EGFR* allelic frequencies. Additionally, a third Quantitative Multiplex FFPE Reference Standard (Horizon Diagnostics) was used. This standard covered mutations at codons 719 (p.G719S), 746–750 (A746-E750del), 790 (p.T790M), 858 (p.L858R), with mutant *EGFR* allelic frequencies of 24.5, 2, 1 and 3%, respectively.

#### Statistical analysis

Statistical analysis was performed using Fisher's exact or χ^2^ tests. P<0.05 was considered to indicate a statistically significant difference between values. Statistical analysis was performed with the MedCalc software v.12.7.2 (MedCalc Software bvba, Ostend, Belgium).

## Results

### 

#### Sensitivity tests

**Figure 1.**
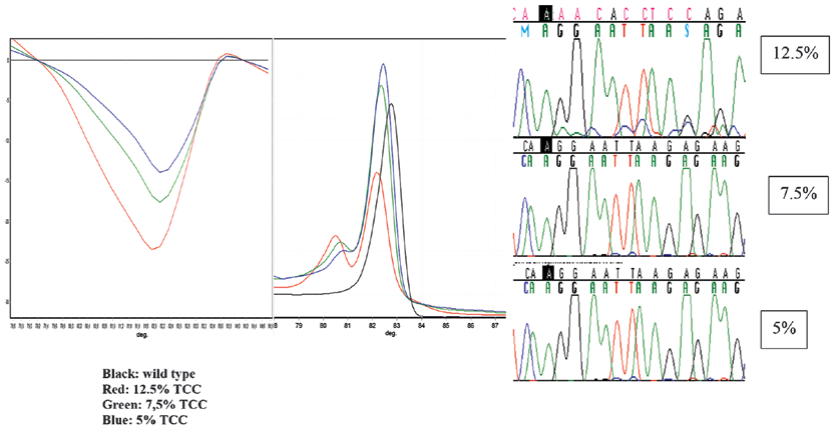
Difference graph, melting curves and sequencing chromatograms of the sensitivity test for the A746-E750del mutation in exon 19 of the *epidermal growth factor receptor* gene. Serial dilutions were performed in order to obtain a mutant to wild-type allele ratio of 12.5, 7.5 and 5%.

Using HRM, it was determined that the mutant EGFR A746-E750del allele frequency in wild-type DNA was 5% and mutant p.L858R allele frequency in wild-type DNA was 7.5% (Table II). Using the Sanger sequencing method for the same mutations, 12.5% mutant alleles was detected in wild-type DNA (Fig. 1). The NGS methodology used detected the A746-E750del mutation with a sensitivity of 2%, the p.L858R mutation with a sensitivity of 3%, while for p.L861Q, p.T790M and p.G719S, a mutant allele frequency of 5% was detectable (Table II).

#### EGFR mutation detection methods

A mutation in exons 18, 19, 20 or 21 of the EGFR gene was detected in 15.83% (233/1,472) of the patients. In 1,239 patients (of the 1,472 tested), no mutation in the *EGFR* gene was detected using both HRM and sequencing (Table II). Of note, there was a 99.8% concordance between the HRM method and Sanger sequencing. HRM technology has been reported to be more sensitive than sequencing (19); however, this method may only be used for mutation screening, not for mutation characterization, thus an alternative NGS method was developed and validated. The accuracy of the NGS technology was determined through analyzing 30 samples with known *EGFR* mutations and 30 samples normal for the EGFR gene, which carried KRAS exon 2 mutations. The samples with an *EGFR* mutation included 20 samples with EGFR exon 19 deletions, 1 sample with an exon 20 insertion, 1 sample with the T790M mutation in exon 20, 2 samples with the G719S mutation in exon 18, 4 samples carrying the point mutation L858R in exon 21 and 2 samples with the L861Q exon 21 mutation. The results revealed a 100% concordance with the HRM method.

In 3 cases, an abnormal melting profile was observed using HRM, while no mutation was detected using Sanger sequencing analysis. These cases all concerned exon 19 amplicons. Since the sensitivity of HRM was found to be superior compared to sequencing, it was proposed that these samples contained a low percentage of mutant alleles, which could not be detected by sequencing. To test this hypothesis NGS was used as an alternative method for mutation detection. The results confirmed the presence of a deletion mutation in exon 19 of the *EGFR* gene in all 3 samples. All three detection methods used exhibited a high specificity, as no false positive samples were detected in the 1,239 normal samples tested. However, Sanger sequencing did not detect 3/233 positive samples (1.29%), indicating that this method exhibits a decreased level of sensitivity compared with the HRM and NGS methods (Table II).

#### EGFR mutation distribution and patient characteristics

**Figure 2.**
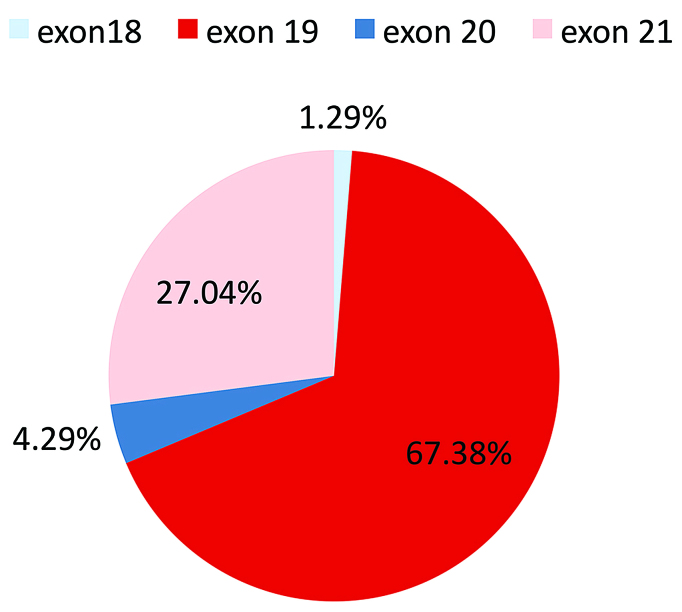
*Epidermal growth factor receptor* mutation spectrum in Greek non-small-cell lung cancer patients. Percentages were calculated out of the total of mutated tumors.

In this population of Greek patients it was demonstrated that the *EGFR* mutation distribution was 3 in exon 18 (1.29%), 157 in exon 19 (67.38%), 10 in exon 20 (4.29%) and 63 in exon 21 (27.04%) (Fig. 2). All mutations detected in exon 19 were deletions. The most common mutation was the A746-E750del in exon 19 (76.43% of exon 19 mutations). The p.L858R mutation was the dominant mutation in exon 21, accounting for 90% of the mutations detected.

Out of the 1472 Greek patients, 1,077 (73%) were male and 395 (27%) were female. The mutation percentages were 11.96 (126/1,077) and 27.09% (107/395) for males and females, respectively. The mean age of diagnosis was 63 years. The majority of tumors with known histology were adenomas (82.49%). In addition, ~73% (409/561) of patients with known smoking status were smokers or ex-smokers (78% of males and 54% of females) (Table III).

#### Association of patient characteristics with EGFR mutation frequency

There were notable differences in EGFR mutation frequency between male and female patients. In male patients the mutation frequency was lower than in female, indicating a gender-associated EGFR mutation frequency (P<0.001) (Table IV).

*EGFR* mutation rate was more prevalent in the non-smoker group compared with the ex-smoker and smoker groups (25.66% vs. 13.33 and 11.08%, respectively; P=0.0002). However, these values were almost equivalent for males (10.00 vs. 11.11 and 10.46%, respectively; P=0.9759), while a significant difference in the mutation percentage was observed between female non-smokers and ex-smoker or smokers (48.39 vs. 25.00 and 13.16% respectively; P<0.0001) (Table IV).

These results indicated that Greek female non-smokers were more likely to present with an *EGFR* mutation, while the mutation percentage in males was substantially lower and independent of cigarette smoking.

Histology of the tumors was observed to be associated with mutation rates (P=0.6707, males; P=0.0424, females; P=0.0313, both genders). The greatest mutation percentage was observed in adenosquamous tumors (35.71%) (Table IV); however, the low number of samples with this type of tumor does not allow for conclusions to be drawn. By contrast, squamous NSCLC were associated with reduced mutation rates in males and females.

*EGFR* mutation frequency was comparable in patients of age range 40–60 and 60+, while mutation rates were higher in those under the age of 40 (Table IV). Comparable results were observed between males and females <40 years (Table IV); however the low number of younger patients with NSCLC does not allow for conclusions to be drawn.

#### KRAS exon 2 mutation frequency

A KRAS exon 2 mutation was observed in 18.89% (106/561) of all tumors analyzed. The majority of mutations were observed in codon 12 (90/106, 84.90%). No difference in the mutation frequency was observed in the mutation frequencies between smokers, ex-smokers or non-smokers; this was the case for both genders (Table V).

The distribution of different *KRAS* mutations among the different groups is represented in Table VI. Of note, the majority of *KRAS* mutations detected in the smoker and ex-smoker groups (81.54 and 100%, respectively) were transversion mutations (substitution of a purine for a pyrimidine or conversely, G→T or G→C), which are known to be smoking-associated (21,22). By contrast, the percentages of transversion and transition mutations (substitution of a purine for a purine, e.g., G→A or a pyrimidine for a pyrimidine, C→T) were equally distrubted in the non-smoker group (57.14% transversion mutations; 42.86% transition mutations).

## Discussion

In the present study, HRM analysis was performed in order to produce specific melting profiles for distinguishing between wild-type and mutated *EGFR* and *KRAS* genes in patient samples. All mutations detected through Sanger sequencing were also analyzed using HRM. The present study confirmed that the HRM was highly sensitive, as mutant/wild-type allele detection was achieved between 5 and 7.5%, dependent on the mutation type and amplicon; whereas Sanger sequencing analysis had a sensitivity of 12–15%. It was previously reported that Sanger sequencing was a less sensitive method compared with HRM analysis; however, HRM analysis has also been demonstrated to produce false positive results due to bad DNA quality, this most commonly occurs when the starting material is FFPE tissue (19,20). In addition, although the HRM method may be used as a screening method for mutation detection, it cannot characterize the mutation detected. In the present study, 3 cases that concerned the *EGFR* exon 19 amplicon were found to be positive for a mutation using HRM, whereas the Sanger sequencing found these cases to be negative. Therefore, despite the higher sensitivity of HRM analysis compared with that of the Sanger sequencing method, an alternative NGS method was used to confirm the presence of a mutation. The NGS method is able to detect 2–5% of mutant alleles, as it was proved for p.L861Q, 746–750del, p.L858R, p.T790M and p.G719S mutations in the present study. In addition, NGS is a rapid, sensitive and accurate method for somatic mutation detection. It requires minimum manual input and is able to detect different somatic mutations simultaneously. However, it requires DNA of very good quality and quantity (>10 ng/µl), which is sometimes difficult to obtain when using FFPE tissues (23,24). Furthermore, NGS is more expensive compared with HRM and Sanger sequencing when a small amount of samples (<30) is processed in a single experiment. Thus, it is a superior method whenever the analysis of >1 gene is required and/or >50 samples are processed in the same experiment.

Another important factor affecting the sensitivity of mutation detection is appropriate tissue selection. Therefore, the present study considered the existence of pathological review crucial for all samples, in order to ensure a tumor cell content of >75%.

In the present study, 1,472 patients were subjected to *EGFR* mutation screening. The *EGFR* mutation rates have previously been reported to differ largely among different populations (12). In the present Greek population the overall *EGFR* mutation frequency was 15.83%.

The *EGFR* mutation frequency in Caucasian NSCLC patients was reported to be 10–15% (12). A recent study reviewed *EGFR* mutation incidence in European countries (25). *EGFR* mutation frequency in Europe ranged from 6% (Switzerland) to a maximum of 37.5% (Germany), depending on ethnicity and patient's characteristics. In a previous study, a low percentage of *EGFR* mutations (8.2%) was observed in a Greek population; however, in the present study, a higher percentage of *EGFR* mutation frequency was observed. This may be due to the larger number of patients analyzed in the present study or to different clinicopathological characteristics of this cohort. For example, 82.49% of the patients analyzed in the present study were diagnosed with adenocarcinomas, which are known to present greater percentage of *EGFR* mutation rates compared to other types of NSCLC.

Of the 1,472 patients tested in the present study, 73% were males and 27% were females. As expected, differences in *EGFR* mutation frequency were observed between male and female patients. In male patients the mutation frequency was lower compared with females, indicating a gender-associated *EGFR* mutation frequency (P<0.001).

In the present study, >72% of patients with known smoking status were smokers or ex-smokers (78% of males and 54% of females). This indicated that as previously reported, smoking is an important factor in NSCLC etiology (3,4).

A previous study investigating the impact of cigarette smoking on cancer risk in the European population indicated that the hazard ratio of developing lung cancer for smokers was 23.30 for men and 7.53 for women (2). This indicates that the risk of NSCLC is slightly higher for men compared with women. This may be due to different male-female habits or differences in the physiology between the two genders. In European countries, almost 1 in every 5 cancers is caused by cigarette smoking (3). Using data on cancer incidence for 2008, it was estimated that in Greece 7,653 novel cancer diagnoses per year were attributed to cigarette smoking (2).

In the present study, when smoking habits are taken in consideration it was revealed that, in females the mutation frequency differs substantially between non-smokers, ex-smokers and smokers (P=0.001). By contrast, *EGFR* mutation frequency was comparable between non-smoking and smoking males. Female smokers demonstrated a comparable mutation frequency to that of males. This indicated that the mechanism of tumor development differs substantially in females depending on their smoking habits.

Additionally, the present study demonstrated that *EGFR* mutation rates were higher in adenoma and adenosquamous NSCLC compared with squamous NSCLC (P=0.001). *EGFR* mutation frequency was comparable in patients aged 40–60 years and >60 years, while mutation rates were higher in those aged <40 years. Comparable results were observed between males and females <40 years; however the low number of younger NSCLC patients does not allow for conclusions to be drawn.

Concerning *KRAS* mutation frequency, numerous studies have suggested that it is significant lower in non-smokers compared to smokers (16,21,22). However, these conclusions were not observed in the present study population. In accordance with previous studies, a different mutation distribution was observed between non-smokers, ex-smokers and smoker groups (16,22). The prevalence of transversion mutations in ex-smokers and smokers is in accordance with previous studies and suggested the association of these mutations with smoking (16,22). Whereas *KRAS* transitions mutations were more common in lung adenocarcinomas from patients without any smoking history.

In conclusion, to the best of our knowledge, the present study was the largest study reporting *EGFR* mutation spectrum and frequencies in a cohort of Greek NSCLC patients. Sensitive mutation detection techniques were used in routine diagnostic practice in order to obtain an overall mutation frequency of 15.83%. In addition, these results demonstrated that female non-smokers had a high prevalence of *EGFR* mutations. Furthermore, *KRAS* mutation analysis in patients with known smoking history revealed no difference in mutation frequency according to smoking status, although a higher prevalence of transversion mutations in the ex-smoker and smoker groups was observed. This study highlights the importance of sensitive molecular techniques, such as NGS for *EGFR* and *KRAS* mutation analysis. Furthermore, this technique may be used in future studies for simultaneous mutation analysis of a number of genes that are prognostic and/or predictive markers in lung cancer patients.

## Figures and Tables

**Table I.** Targets for next generation sequencing assay, chromosomal location, length of the amplified regions and number of amplicons per target.

Target	Chromosome: start-stop	Length (bp)	Amplicons
*NRAS*_Exon_2258071±0	1:115, 256, 459–115, 256, 546	88	1/1
*NRAS*_Exon_2255597±0	1:115, 258, 671–115, 258, 798	128	1/1
*NRAS*_Exon_2255265±0	1:115, 252, 190–115, 252, 349	160	2/2
*KRAS*_Exon_2084598±0	12:25, 398, 208–25, 398, 329	122	2/2
*KRAS*_Exon_2081588±0	12:25, 380, 168–25, 380, 346	179	3/3
*KRAS*_Exon_2081272±0	12:25, 378, 548–25, 378, 707	160	2/2
*KIT*_Exon_1948841±0	4:55, 592, 023–55, 592, 216	194	3/3
*KIT*_Exon_1925438±0	4:55, 593, 582–55, 593, 708	127	2/2
*KIT*_Exon_1923956±0	4:55, 594, 177-55, 594, 287	111	1/1
*KIT*_Exon_1923106±0	4:55, 599, 236–55, 599, 358	123	2/2
*HRAS*_Exon_1850745±0	11:533, 441–534, 375	935	11/11
*EGFR*_Exon_2086565±0	7:55, 242, 415–55, 242, 513	99	1/1
*EGFR*_Exon_2085900±0	7:55, 241, 614–55, 241, 736	123	2/2
*EGFR*_Exon_2085577±0	7:55, 248, 986–55, 249, 171	186	3/3
*EGFR*_Exon_2084815±0	7:55, 259, 412–55, 259, 567	156	3/3
*BRAF*_Exon_2290211±0	7:140, 481, 376–140, 481, 493	118	2/2
*BRAF*_Exon_2290058±0	7:140, 453, 075–140, 453, 193	119	1/1

**Table II.** Comparison of Sanger sequencing, HRM and NGS methods used for mutation detection.

	Detection method		
	
Parameters	Sanger sequencing	HRM	NGS
Limit of detection, %^a^	12.50	5.0–7.50	2.0–5.0
Specificity, % (true negative)	100 (1239/1239)	100 (1239/1239)	100 (30/30)
Missed mutations, n (%)	3/233 (1.29)	0/233 (0.00)	0/30 (0.00)
Total samples tested, n	1472	1472	60

a Frequency of mutated alleles detected in a background of wild type alleles. HRM, High-resolution melting curve; NGS, next-generation sequencing.

**Table III.** Patient demographics.

Variables	No. of patients	%
Gender (n=1,472)		
Male	1,077	73.00
Female	358	27.00
Age (n=1,046)		
<40	24	2.29
40–60	384	36.71
>60	638	60.99
Histology (n=497)		
Adenomas	410	82.49
Squamous	62	12.47
Adenosquamous	14	2.82
Large-cell	11	2.21
Smoking status (n=561)		
Smokers	334	59.54
Ex-smokers	75	13.37
Non smokers	152	27.09

**Table IV.** Incidence of *epidermal growth factor receptor* mutation per clinical factor in Greek non-small-cell lung cancer patients.

Clinical factor	Male (%)	Female (%)	Total (%)
Histology			
Adenomas	28/286 (9.70)	40/124 (32.26)	68/410 (16.58)
Squamous	4/54 (7.41)	0/8 (0.00)	4/62 (6.45)
Adeno-squamous	2/10 (20.00)	3/4 (75.00)	5/14 (35.71)
Large cell	1/9 (11.11)	0/2 (0.00)	1/11 (9.09)
P-value	0.6707	0.0424	0.313
Smoking status			
Smokers	27/258 (10.46)	10/76 (13.16)	37/334 (11.08)
Ex-smokers	7/63 (11.11)	3/12 (25.00)	10/75 (13.33)
Non-smokers	9/90 (10.00)	30/62 (48.39)	39/152 (25.66)
P-value	0.9759	<0.0001	0.0002
Age, years			
23–40	3/14 (21.43)	2/10 (20.00)	5/24 (20.83)
40–60	28/274 (10.22)	29/110 (26.36)	57/384 (14.84)
60+	52/468 (11.11)	49/170 (28.82)	101/638 (15.83)
P-value	0.4201	0.7785	0.7074
None	126/1,077 (11.69)	107/395 (27.09)	233/1,472 (15.83)

**Table V.** *Kirsten-rat sarcoma oncogene homolog* exon 2 mutation frequency according to gender and smoking history.

	Smoking status			
		
Gender	Smokers (%)	Ex-smokers (%)	Non smokers (%)	P-value
Male	50/258 (19.38)	11/63 (17.46)	18/90 (20)	0.9207
Female	15/76 (19.73)	2/12 (16.67)	10/62 (16.12)	0.8535
Total	65/334 (19.46)	13/75 (17.33)	28/152 (18.42)	0.8997

**Table VI.** Distribution of *KRAS* mutations according to smoking history.

Type of *KRAS* mutation	Smokers (%)	Ex-smokers (%)	Non-smokers (%)
Transversion mutation			
c.35G>T (p.G12V)	26/65 (40)	6/13 (46.15)	10/28 (35.71)
c.34G>T (p.G12C)	18/65 (27.69)	5/13 (38.46)	5/28 (17.86)
c.37G>T (p.G13C)	9/65 (13.85)	2/13 (15.38)	1/28 (3.57)
Total	53/65 (81.54)	13/13 (100)	16/28 (57.14)
Transition mutation			
c.35G>A (p.G12D)	10/65 (15.38)	0/13/(0)	9/28 (32.14)
c.38G>A (p.G13D)	2/65 (3.08)	0/13/(0)	2/28 (7.14)
c.34G>A (p.G12S)	0/65 (0)	0/13/(0)	1/28 (3.57)
Total	12/65 (18.46)	0/13 (0)	12/28 (42.86)

KRAS, Kirsten-rat sarcoma oncogene homolog.
